# Advances in Hyperbaric Oxygen Therapy: Medical Benefits and Technical Perspectives

**DOI:** 10.1007/s10439-026-04027-7

**Published:** 2026-02-18

**Authors:** Antoanela Naaji, Monica Ciobanu, Marius Popescu

**Affiliations:** Department of Economic and Technical Sciences, Vasile Goldis Western University of Arad, Arad, Romania

**Keywords:** Hyperbaric oxygen therapy, Pressurized medical chamber, Modeling technique, General equations of hydraulics, Hydraulic parameters, Laboratory model

## Abstract

In the rapidly evolving landscape of medicine, hyperbaric oxygen therapy (HBOT) has emerged as a clinically recognized treatment involving the inhalation of pure oxygen in a pressurized chamber. Despite its proven applications, further research is needed to understand and simulate the physical processes governing HBOT. This paper presents a novel modeling technique and an automated pressurized chamber specifically designed for laboratory studies to better analyze oxygenated air circulation in hyperbaric environments.

The proposed model integrates hydraulic principles and geometric constraints to replicate real-world HBOT dynamics. It incorporates dimensionless equations, including Reynolds, Froude, and Archimedes principles, to account for fluid motion, energy dissipation, and pressure field behavior. Geometric conditions involve initial and boundary parameters such as velocity, temperature, pressure, concentration, and mass density. For realistic simulation, both physical and geometric similarity conditions must be satisfied. To enhance the generalizability of results, the Ruark transformation is employed to introduce dimensionless coordinates, allowing findings to extend to related scenarios.

The proposed laboratory model demonstrates the ability to accurately simulate complex oxygenation and flow dynamics in pressurized environments. The automated chamber ensures precise control and experimental reproducibility. The model effectively reproduces velocity fields and pressure distributions across varied geometric and dynamic configurations.

By combining hydraulic theory with geometric modeling, this study provides a robust framework for exploring HBOT mechanisms in a controlled setting. The approach not only advances theoretical understanding but also lays the groundwork for future experimental and clinical investigations in hyperbaric therapy and similar therapeutic environments.

## Introduction

Hyperbaric oxygen therapy (HBOT) is a well-established medical procedure involving the inhalation of oxygen at pressures exceeding normal atmospheric levels. Originally developed to support divers experiencing decompression-related injuries, HBOT has since evolved into a recognized therapeutic practice within hospital and specialized clinical units [1–5].

Current clinical guidelines issued by the Hyperbaric Medical Society identify a defined list of approved conditions for HBOT, such as decompression sickness, arterial gas embolism, severe carbon monoxide poisoning, and specific types of radiation injury or non-healing wounds [6–9].

According to the Undersea and Hyperbaric Medical Society (UHMS), hyperbaric oxygen therapy is approved for more than fifteen medical indications. These include air or gas embolism, decompression sickness, carbon monoxide poisoning, clostridial myonecrosis (gas gangrene), crush injury, compartment syndrome, radiation-induced tissue injury, refractory osteomyelitis, necrotizing soft tissue infections, diabetic foot ulcers, compromised skin grafts and flaps, severe anemia, intracranial abscess, thermal burns, and sudden sensorineural hearing loss. These well-established clinical applications highlight the therapeutic breadth of HBOT and emphasize the importance of precise oxygen and pressure regulation within hyperbaric systems.

During HBOT, the ambient pressure within the chamber is typically elevated to two or three atmospheres, substantially enhancing the amount of oxygen dissolved directly in plasma and, consequently, increasing the oxygen diffusion gradient toward hypoxic tissues.

The physiological effects of HBOT are driven by several gas laws and biochemical responses. Elevated pressure increases plasma oxygen concentration by an order of magnitude, facilitating oxygen diffusion into hypoxic or poorly perfused tissues [10–14].

Hyperoxia can stimulate angiogenesis, modulate inflammatory signaling, influence mitochondrial respiration, and activate several cellular repair pathways. Recent studies increasingly demonstrate the therapeutic relevance of HBOT for neurological and systemic disorders, including traumatic brain injury, neuroinflammation, ischemia–reperfusion injury, and cognitive dysfunction [15–20]. These studies underscore the complex physiological effects associated with hyperoxic exposure and provide important context for understanding how chamber dynamics influence therapeutic delivery.

Complementing these findings, additional investigations have examined the antimicrobial and immunomodulatory roles of hyperbaric oxygen. HBOT has been shown to enhance leukocyte activity, promote bacterial susceptibility to oxidative stress, and support tissue recovery in difficult-to-heal wounds and infections [21–30]. Research into regenerative medicine, vasculogenesis, and adjunctive oncological applications has further broadened scientific interest in HBOT. While these areas remain under clinical evaluation and have not yet reached universal therapeutic validation, they highlight the multifaceted systemic responses triggered by changes in pressure and oxygenation.

From an engineering viewpoint, HBOT chambers operate as controlled thermodynamic and fluid-dynamic systems in which pressure, temperature, oxygen concentration, and airflow interact continuously. The design and optimization of these chambers require a precise understanding of gas flow behavior, pollutant dispersion, thermal gradients, and pressure regulation. Mathematical modeling is therefore critical to improving chamber performance and ensuring safe operation. Dimensionless analysis, using Reynolds, Froude, Archimedes, Peclet, and related numbers, provides a systematic approach to describing fluid motion and heat or mass transfer under pressurized conditions [31–36]. Previous studies have demonstrated the value of analytical and computational modeling in predicting oxygen diffusion, pressure stability, and flow distribution in enclosed spaces [37–39]. However, experimental validation in scaled physical setups remains limited, and the correspondence between theoretical predictions and real chamber dynamics has yet to be systematically evaluated.

In HBOT systems, pressure regulation represents one of the most important safety and performance parameters. Chamber pressurization and depressurization rely on coordinated interactions between pumps, solenoid valves, pressure sensors, and control algorithms embedded within microcontroller or computer-based systems [40–46]. The dynamic behavior of these components determines treatment stability, energy efficiency, and safety under varying operational phases. Accurate regulation must account for gas compressibility, valve flow coefficients, chamber capacity, and transient response times. Energy dissipation due to thermodynamic irreversibility, heat transfer between incoming and resident gases, and pollutant dilution further contribute to the complexity of HBOT chamber behavior.

The present study addresses these challenges by integrating mathematical modeling, laboratory-scale experimentation, and automated control system design into a unified analytical framework. First, we develop a dimensionless mathematical model describing the dynamics of oxygenated air circulation in a hyperbaric environment. This includes the derivation of continuity, Navier–Stokes, thermal energy, and mass diffusion equations, together with similarity conditions and geometric constraints. Second, we design a scaled-down hyperbaric chamber that adheres to applicable similarity principles and enables controlled evaluation of airflow and pressure dynamics. Third, we implement a prototype pressure regulation system based on solenoid valve actuation, pump control, and an embedded microcontroller platform equipped with a graphical interface for real-time monitoring. Finally, we conduct a series of laboratory experiments to evaluate the system’s performance in terms of pressure stabilization, valve response, and airflow behavior within the scaled hyperbaric chamber model.

It is important to emphasize that this study does not include clinical testing, biological assays, or direct assessment of patient outcomes. All clinical references represent previously published findings, whereas the present work focuses strictly on the engineering aspects of HBOT chamber behavior. By combining theoretical modeling with controlled laboratory experimentation, this study aims to contribute to the systematic design, validation, and optimization of hyperbaric chamber technologies. The methodology presented here may serve as a foundation for future developments, including computational fluid dynamics (CFD), oxygen concentration mapping, sensor integration, and translational studies evaluating chamber design in clinical environments.

Despite the clinical effectiveness of HBOT, current hyperbaric systems still face several technical and operational challenges. These include limited automation of pressure and oxygen control, delayed feedback in mechanical regulation loops, and variability in chamber conditions that can affect treatment reproducibility. Moreover, most existing systems rely on empirical calibration rather than predictive modeling of gas flow and pressure dynamics. The motivation of this study is to address these limitations through a mathematical and experimental framework capable of accurately simulating and controlling oxygen and pressure variations, thereby enhancing both safety and therapeutic precision.

To address these limitations, the present research proposes a multidisciplinary modeling and control approach. From a biomedical perspective, precise regulation of pressure and oxygen dynamics in hyperbaric oxygen therapy directly influences tissue oxygenation and metabolic stability. Controlled pressurization minimizes oxidative stress while ensuring optimal oxygen delivery to hypoxic tissues. Stable decompression cycles reduce the risk of barotrauma and microbubble formation, thereby improving patient safety and comfort. Consequently, engineering precision in HBOT systems translates into measurable physiological and therapeutic benefits, including enhanced wound healing, angiogenesis, and recovery in ischemic or radiation-injured tissues.

Following the current state of hyperbaric medicine and related research (Section “Current State of Hyperbaric Medicine and Related Research”), Section “Methodology” presents the methodology used in this study. Section “Mathematical Modeling of Oxygenated Air Dynamics in Hyperbaric Chambers” outlines the mathematical model describing air recirculation in hyperbaric chambers and defines the dimensional and functional parameters for the scaled laboratory setup. Section “Structure and Function of the Pressure Regulation System in Hyperbaric Chambers” details the structure and operation of the pressure regulation system, while Section “Laboratory implementation and testing of the pressure regulation system” describes its laboratory implementation and experimental validation. The final section provides the conclusions and perspectives for future work.

## Current State of Hyperbaric Medicine and Related Research

### Hyperbaric Oxygen Therapy

Within the current landscape of hyperbaric medicine, hyperbaric oxygen therapy (HBOT) has emerged as a pivotal therapeutic intervention across a wide spectrum of medical conditions, characterized by the inhalation of pure oxygen in a pressurized chamber.

The efficacy of HBOT in treating conditions such as decompression sickness, arterial embolism, and severe carbon monoxide poisoning is well documented [7–9]. This therapy enhances the oxygen-carrying capacity of blood, promoting oxygenation in ischemic tissues and supporting anti-inflammatory and anti-infective processes [25]. Research has further demonstrated its utility in managing complex medical scenarios, including traumatic brain injuries (TBI) and post-traumatic stress disorder (PTSD), with studies indicating improved neural healing and axonal growth under hyperbaric conditions [15–17].

Beyond its established applications, HBOT is increasingly recognized for its potential in adjunctive therapies. For example, it has shown promise in enhancing the efficacy of chemotherapy and radiation therapy for cancer patients without promoting tumor growth [26, 27]. Its role in mobilizing stem cells and promoting angiogenesis underscores its potential in regenerative medicine [28]. Additionally, HBOT’s capacity to elevate tissue oxygen levels significantly contributes to its effectiveness in chronic wound management and infection control [29, 30].

Recent advancements have focused on integrating HBOT with computational modeling to optimize its application. Mathematical models have been employed to simulate oxygen diffusion and pressure dynamics within hyperbaric chambers, providing insights that enhance patient safety and treatment efficacy [31, 32]. These innovations underscore the importance of precision and adaptability in HBOT protocols, particularly in tailoring treatments to individual patient needs.

Hyperbaric oxygen therapy (HBOT) has emerged as a pivotal therapeutic intervention across a spectrum of medical conditions, characterized by the inhalation of pure oxygen in a pressurized chamber. Its historical development is deeply rooted in addressing the health challenges faced by divers and has since expanded to encompass numerous clinical applications.

Key features and benefits of HBOT include the following:

1. Enhanced Oxygen Diffusion: HBOT facilitates the diffusion of oxygen to damaged or poorly vascularized tissues, promoting healing through increased oxygen availability in body fluids.
2. Neovascularization and Stem Cell Stimulation: The therapy stimulates angiogenesis (formation of new blood vessels) and vasculogenesis (formation of new vascular structures), as well as the production of natural stem cells. These processes aid in tissue restoration and accelerate the healing of wounds and injuries.
3. Immune System Enhancement: HBOT activates lymphocytes, bolstering the immune system’s ability to combat infections caused by viruses, aerobic bacteria, and anaerobic bacteria. This immunomodulatory effect is critical in enhancing the body’s natural defenses.
4. Osteogenesis Stimulation: The therapy stimulates bone formation (osteogenesis) and significantly reduces healing time for fractures and osteoarticular traumas, making it beneficial for orthopedic applications.
5. Collagen and Elastin Production: By increasing the synthesis of collagen fibers and natural elastin, HBOT contributes to visible anti-aging effects, such as wrinkle reduction and improved skin elasticity.
6. Cellular Oxygenation and Oxidative Stress Reduction: HBOT boosts cellular oxygenation and mitigates oxidative stress, which is beneficial in managing conditions associated with hypoxia and inflammation.
7. Antibacterial Effects: The therapy exhibits potent antibacterial properties, and in certain cases, HBOT is the only effective treatment for infections caused by polyresistant bacteria.

These advanced technological and biological effects underscore the multifaceted therapeutic potential of HBOT, making it a valuable treatment modality in both clinical and emergency settings.

### Cutting-Edge Technologies in Hyperbaric Oxygen Therapy

Hyperbaric oxygen therapy (HBOT) significantly enhances the transport and availability of oxygen within the body. Under normal atmospheric conditions, approximately 97% of oxygen in the blood is bound to hemoglobin, while the remaining portion is dissolved in plasma. During HBOT, as per Henry’s law, the amount of dissolved oxygen increases substantially, leading to improved tissue oxygenation. This elevated level of oxygen in solution can reach areas that are otherwise inaccessible to red blood cells, providing critical oxygenation in cases of severe anemia or carbon monoxide poisoning [33].

HBOT also promotes the production of reactive oxygen species (ROS), which play a crucial role in cellular processes. These ROS oxidize membrane proteins and lipids, disrupt DNA integrity, and inhibit bacterial metabolic functions, thereby contributing to the therapy’s antibacterial effects [34, 35]. Specifically, HBOT is highly effective against anaerobic bacteria and enhances the activity of oxygen-dependent peroxidases, boosting leukocyte-mediated bacterial destruction. Moreover, HBOT improves the penetration of certain antibiotics through bacterial cell walls, increasing their efficacy [36].

### Research and Clinical Studies on HBOT Efficacy

Research has shown that hyperbaric oxygen therapy (HBOT) mobilizes bone marrow-derived stem cells into systemic circulation, facilitating their distribution throughout the body [37]. Studies involving human subjects have demonstrated that these stem cells can cross the blood-brain barrier and differentiate into neurons, astrocytes, and microglia. This process aids in the repair and regeneration of neural tissue, thereby improving various pathophysiological conditions and enhancing overall health [38, 39].

Regarding cancer treatment, HBOT’s effects are complex and multifaceted. Some studies have suggested that HBOT may promote angiogenesis, which could potentially support tumor growth or recurrence. However, other research indicates that HBOT can have inhibitory effects on tumor progression [40, 41]. Independent research groups have reviewed extensive experimental and clinical data from the past 50 years, concluding that intermittent exposure to HBOT does not stimulate the growth of primary or metastatic cancers. Clinically, HBOT is often used as an adjunct to radiation therapy and chemotherapy in the treatment of malignant tumors, helping to improve treatment outcomes and patient recovery.

The role of HBOT in enhancing treatment outcomes and its safety profile in various therapeutic contexts continues to be an area of active research. Current evidence supports its use in diverse clinical scenarios, ranging from wound healing and infection control to neurological recovery and cancer therapy. As research progresses, the therapeutic applications of HBOT are expected to expand, offering new hope for patients with challenging medical conditions.

## Methodology

This study adopts a multidisciplinary approach to explore the dynamics of hyperbaric oxygen therapy, combining theoretical modeling, experimental validation, and practical implementation. The methodology is structured as follows:

1. The development of mathematical framework in order to understand the oxygenated air circulation within hyperbaric chambers, incorporating fundamental principles of hydraulics, such as Reynolds, Froude, and Archimedes numbers. Dimensionless coordinates, introduced via the Ruark transformation, were employed to extend the applicability of findings to analogous scenarios.
2. The design of laboratory models by constructing a scaled-down hyperbaric chamber to replicate real-world conditions. The model adhered to geometric similarity principles, ensuring the accurate representation of velocity fields, pressure distributions, and associated forces. The chamber’s dimensions and parameters were optimized to simulate oxygen diffusion and energy dissipation effectively.
3. The implementation of a pressure regulation system by using a combination of solenoid valves and pumps. The system’s operation was governed by an ATMEL AT89C52 microcontroller, interfaced with a graphical user interface developed in LabWindows. This setup facilitated real-time monitoring and precise adjustments to chamber pressure.
4. Experimental validation of the model, by using a series of tests to validate the theoretical predictions. Parameters such as oxygen concentration, pressure dynamics, and thermal effects were measured and compared against the model’s outputs. These experiments also assessed the feasibility of the pressure regulation system under varying operational conditions.
5. Data Analysis and Interpretation in order to refine the mathematical model and optimize treatment protocols. Statistical methods were applied to evaluate the correlation between model predictions and observed outcomes, ensuring the reliability of the findings.

This comprehensive methodology bridges theoretical insights with practical applications, providing a robust framework for advancing the efficacy and safety of hyperbaric oxygen therapy.

This research did not involve experiments with human participants or live animals. All procedures were limited to mathematical modeling, laboratory-scale physical simulations, and technical validation of the pressure control system under controlled conditions.

**Fig. 1:**
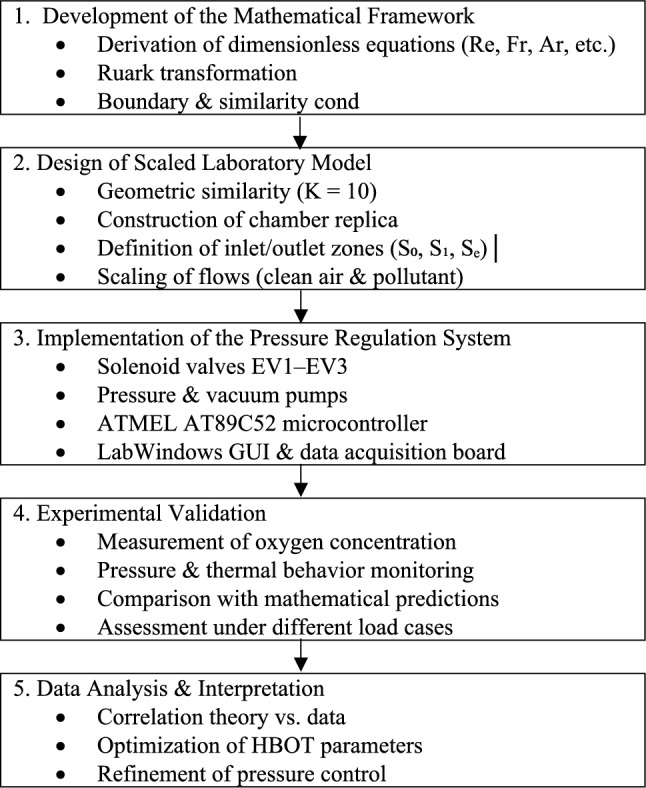
Flowchart describing the methodology applied in developing, simulating, implementing, and validating the hyperbaric oxygen therapy model and laboratory system

To provide a concise overview of the methodological approach, Figure 1 presents the workflow adopted in this study. The diagram highlights the main stages of the research, including the development of the mathematical model, the design of the scaled laboratory chamber, the implementation of the pressure regulation system, and the subsequent experimental validation and data analysis. This visual representation supports a clearer understanding of how the different components of the methodology are integrated.

## Mathematical Modeling of Oxygenated Air Dynamics in Hyperbaric Chambers

Mathematical models provide a systematic framework for understanding and predicting the behavior of complex systems. In the context of hyperbaric chambers, they allow researchers and clinicians to simulate different scenarios, optimize treatment protocols, and design more effective chambers. These models can describe how oxygen diffuses and circulates within the chamber, how pressure changes over time, and how these factors interact with the patient’s physiology.

One of the primary advantages of mathematical modeling is the ability to conduct virtual experiments. By adjusting parameters such as pressure, oxygen concentration, and treatment duration, models can predict outcomes without the need for extensive clinical trials. This not only saves time and resources but also enhances patient safety by identifying potential risks before implementation in real life [42].

Furthermore, mathematical models help in understanding the underlying mechanisms of HBOT at a cellular and molecular level. For instance, they can elucidate how increased oxygen levels influence cellular metabolism, promote angiogenesis, and aid in the healing of tissues. Such insights are invaluable for developing targeted therapies and improving clinical outcomes.

The primary objective of the mathematical model for oxygenated air dynamics in hyperbaric chambers is to provide a detailed and accurate representation of the physical processes occurring within the chamber. By simulating oxygen distribution, pressure dynamics, and thermal effects, the model provides valuable insights that can improve treatment protocols and patient outcomes. Through continuous validation and patient-specific adaptations, the model ensures that HBOT remains a safe and effective therapeutic option.

### Model Equations

Considering $\vec{i}$ and $\vec{j}$ the unit vectors of the abscissa Ox and the ordinate Oy, then the displacement, speed, time, temperature, dynamic pressure, viscosity, thermal conductivity and concentration in the dimensionless coordinates are [41–43]:

1 $$x_{i} \, = \,\frac{{x_{i} }}{A},\,x_{j} \, = \,\frac{{x_{j} }}{A},\,v_{i} \, = \,\frac{{v_{i} }}{{v_{o} }},\,v_{j} \, = \,\frac{{v_{j} }}{{v_{o} }},\,t_{*} \, = \,\frac{1}{{A/v_{o} }},\,\theta_{*} \, = \,\frac{{\theta - \theta_{o} }}{{\theta_{1} - \theta_{o} }}\, = \,\frac{\Delta \theta }{{\Delta \theta_{o} }},\,\rho_{*} \, = \,\frac{\rho }{{\rho_{o} v_{o}^{2} }},\,\eta_{*} \,\,\frac{\eta }{{\eta_{o} }},\,\lambda_{*} \, = \,\frac{\lambda }{{\lambda_{O} }},\,c_{*} \, = \,\frac{{c - c_{o} }}{{c_{1} - c_{0} }},$$

where *A* is the height of the capsule/tank; $x_{i}$*,* $x_{j}$, respectively, $v_{i}$*,* $v_{j}$ are the projections along the axes Ox, Oy of the displacement, respectively, of the velocity vector; $v_{0}$ is the velocity of the fluid (oxygenated air) at the inlet; $\theta_{1}$ is the temperature of the fluid (oxygenated air) entered; $\Delta \theta$ is the temperature difference between the temperature of the fluid (the air exhaled by the patient) polluting $\theta_{1}$ and the temperature of the clean fluid (oxygenated air) $\theta_{0}$ brought at the inlet in stationary regime; $\rho_{0}$ is the density of the clean air brought to the inlet; $\eta_{0}$ is the dynamic reference viscosity; *λ* is the thermal conductivity of the entered clean fluid; $c_{1}$and $c_{0}$ are the concentrations of the pollutant (exhaled air emitted by the patient), respectively, of the cleaned fluid entered (oxygenated air).

In this model, the variable t represents the exposure duration corresponding to each pressurization or decompression phase of the hyperbaric session. Physiologically, it reflects the time-dependent evolution of oxygen diffusion and gas exchange within tissues, which are critical determinants of HBOT efficacy and treatment outcome.

#### Conditions of Similarity

The continuity equation, which expresses the conservation of the flow along the Ox direction, is

2 $$\frac{{\partial \rho_{*} }}{{\partial t_{*} }} + \frac{\partial }{{\partial x_{{i_{{}} }} }}\left( {\rho_{*} v_{{i_{{}} }} } \right) = 0.$$

The dynamic motion equation (*Navier-Stokes*), along the Ox direction, is

3 $$\rho_{*} \left( {\frac{{\partial v_{i*} }}{{\partial t_{*} }} + v_{j*} \frac{{\partial v_{i*} }}{{\partial x_{j*} }}} \right) = - \frac{{\partial \rho_{*} }}{{\partial x_{i*} }} - \rho_{*} \frac{{g \cdot A\partial a_{*} }}{{v_{0}^{2} \partial x_{i*} }} + \frac{{\eta_{0} }}{{\rho_{0} v_{0} }}.$$

With the component of the viscous origin voltage in the Ox direction:

4 $$\zeta_{ij*} = \eta_{*} \left( {\frac{{\partial v_{{i_{{}} }} }}{{\partial x_{{j_{{}} }} }} + v_{j*} \frac{{\partial v_{j*} }}{{\partial x_{{i_{{}} }} }}} \right) - \frac{2}{3}\eta_{*} \frac{\partial v}{{\partial x}},$$

where the first term represents the normal effort, and the second represents the tangential effort (in which the first index shows the axis on which the considered surface is perpendicular and contains the tangential force, and the second indicates the direction of the force).

The thermal energy equation is

5 $$\rho_{*} \frac{{d\theta_{*} }}{{dt_{*} }} = \frac{{v_{0}^{2} }}{{c_{m} \Delta \theta_{0} }}\frac{{d\rho_{*} }}{{dt_{*} }} + \frac{{\eta_{0} }}{{\rho_{0} v_{0} A}}\frac{{v_{0}^{2} }}{{c_{m} \Delta \theta_{0} }}\phi_{*} + \frac{{\lambda_{0} }}{{\eta_{0} c_{m} }}\frac{{\eta_{0} }}{{\rho_{0} v_{0} A}}div\left[ {\lambda_{*} grad \left( {\theta_{*} } \right)} \right],$$

in which $\phi_{*}$ is the power dissipated by thermodynamic irreversibility; and $c_{m}$ is the mass heat of the fluid at constant pressure.

Assuming that *Fourier’s* law applies for the heat transfer

6 $$q = - \lambda grad \theta,$$

where *q* is the heat flux at the crossing of a surface dS (the normal vector at the surface S), then the heat flux along the direction Ox will be $q_{i} n_{i}$ dS.

The molecular diffusion equation of fluid constituents (*Fick’s* law), which expresses the exchange of substance, is

7 $$\frac{{\partial c_{*} }}{{\partial t_{*} }} + div\left( {c_{*} v_{*} } \right) = \frac{B}{{Av_{0} }}div\left[ {grad \left( {c_{*} } \right)} \right] = \frac{B}{v}\frac{v}{{Av_{0} }}div\left[ {grad \left( {c_{*} } \right)} \right],$$

where the kinematic viscosity of the fluid was denoted by , and by *B* the diffusion coefficient.

The equation of the state of the fluid for fluctuations of concentrations and temperature inside the studied volume, in the hypothesis of an independent density of pressure, can be developed in *Taylor* series, retaining only on the first terms

8 $$\rho \left( {\theta_{0} + \Delta \theta , c_{0} + \Delta c} \right) = \rho \left( {\theta_{0} , c_{0} } \right) + \Delta \theta \frac{\partial \rho }{{\partial \theta }} + \Delta c\frac{\partial \rho }{{\partial c}}\, \Leftrightarrow \,\rho = \rho_{0} + \Delta \theta \left( {\frac{\partial \rho }{{\partial \theta }}} \right)_{0} + \Delta c\left( {\frac{\partial \rho }{{\partial c}}} \right)_{0},$$

where $c_{0}$ is the concentration in the pollutant (air emitted by the patient) of the fluid (oxygenated air) brought to the inlet, and $\rho \left( {\theta_{0} , c_{0} } \right) = \rho_{0}$ is the initial density of the fluid.

Taking into account that the term $- \frac{1}{{\rho_{0} }}\left( {\frac{\partial \rho }{{\partial \theta }}} \right)_{0}$ represents the coefficient of thermal expansion and dividing the previous relation with the term $\rho_{0}$, we obtain

9 $$\frac{\rho }{{\rho_{0} }} = 1 - \beta \Delta \theta + \frac{\Delta c}{{p_{0} }}\left( {\frac{\partial \rho }{{\partial c}}} \right)_{0},$$

or in dimensionless coordinates

10 $$\rho_{*} = 1 - \Delta \theta_{*} \Delta \theta_{0} \beta + \frac{{c_{1} - c_{0} }}{{p_{0} }}\left( {\frac{\partial \rho }{{\partial c}}} \right)_{0} c_{*}.$$

On the other hand, the pressure *p* and the density ρ of the fluid in the tank vary along the trajectory depending on the pressure $p_{0}$ and the density of the fluid brought from the inlet, so that

11 $$\rho \, = \,\rho_{o} \, + \,\rho^{\prime},\,p\, = \,p_{o} \, + \,p^{\prime}\,and\,p_{o} \, = \, - \rho_{o} gA,$$

where *g* represents gravitational acceleration.

The ratio derivative is developed in *Taylor* series (because it intervenes in the equation of dynamic motion), according to the direction Ox of fluid displacement:

12 $$\frac{1}{\rho }\frac{\partial p}{{\partial x_{i} }} = \frac{1}{{\rho_{0} }}\frac{{\partial p_{0} }}{{\partial x_{i} }} + \frac{1}{{\rho_{0} }}\frac{{\partial p^{\prime}}}{{\partial x_{i} }} + \frac{{\rho^{\prime}}}{{\rho_{0}^{2} }}\left( {\frac{{\partial p_{0} }}{{\partial x_{i} }}} \right).$$

Taking into account the previous expression of $p_{0}$, we obtain

13 $$\frac{1}{\rho }\frac{\partial p}{{\partial x_{i} }} = - g\frac{1}{{\rho_{0} }}\frac{\partial h}{{\partial x_{i} }} + \frac{1}{{\rho_{0} }}\frac{{\partial p^{\prime}}}{{\partial x_{i} }} - \left[ {\beta \Delta \theta - \frac{c}{{\rho_{0} }}\left( {\frac{\partial \rho }{{\partial c}}} \right)_{0} } \right]g\left( {\frac{\partial A}{{\partial x_{i} }}} \right)$$

with the normalized shape

14 $$\frac{1}{{\rho_{*} }}\frac{{\partial p_{*} }}{{\partial x_{i*} }} = - \frac{gA}{{v_{0}^{2} }}\frac{{\partial a_{*} }}{{\partial x_{i*} }} + \frac{{\partial p^{\prime}}}{{\partial x_{i*} }} - \left[ {Ar\Delta \theta_{*} - \frac{{c_{1} - c_{0} }}{Fr}\left( {\frac{\partial \rho }{{\partial c}}} \right)_{0} c_{*} } \right]\frac{{\partial A_{*} }}{{\partial x_{i*} }},$$

where noted with $Ar = \Delta \theta_{0} \Delta \beta \frac{gA}{{v_{0}^{2} }}$, the *Archimedes* number (expresses the ratio between the mass forces and the impulse of the fluid jet introduced in the tank) and with $Fr = \frac{{v_{0}^{2} }}{gA}$, the *Froude* number (expresses the similarity in the conditions in which the gravitational forces predominate).

In this context, the dynamic equation of motion has a dimensionless form

15 $$\frac{{\partial v_{{i_{{}} }} }}{{\partial t_{*} }} + v_{{j_{{}} }} \frac{{\partial v_{{i_{{}} }} }}{{\partial x_{{j_{{}} }} }} = - \frac{{\partial p_{*}{\prime} }}{{\partial x_{{i_{{}} }} }} + \left[ {Ar\Delta \theta_{*} - \frac{{c_{1} - c_{0} }}{{Fr \rho_{0} }}\left( {\frac{\partial \rho }{{\partial c}}} \right)_{0} c_{*} } \right]\frac{{\partial a_{*} }}{{\partial x_{{i_{{}} }} }} + \frac{1}{{\mathrm{Re}}}\frac{{\partial \zeta_{ij*} }}{{\partial x_{j} }},$$

where the *Reynolds* number (Re) is given by the ratio $\frac{{\rho v_{0} A}}{{\eta_{0} }}$, which is also applicable in this case, as the speed in the tank is low (air compressibility is neglected).

It is observed that the continuity equation remained unchanged as a form, and in the equation of motion appeared a series of dimensionless quantities (called numbers or criteria), formed with quantities *A*, $v_{0}$, $p_{0}$, $c_{0}$, ... , characteristic of the studied phenomenon.

#### Particular Conditions of the Oxygenation Phenomenon

If it is assumed that the introduced fluid has zero concentration ($c_{0} = 0$), and then in the new configuration, the boundary conditions are written:

- for speeds: $\vec{v} = \vec{0}$ on the wall surfaces (either $\vec{v}_{*} = \vec{0}$ on the tank wall surfaces); $\vec{v} = \frac{1}{{S_{0} }}\left( {D - D_{1} } \right)\vec{i}$ at the inlet (either $\vec{v}_{*} = \vec{i}$ at the inlet in the tank); $\vec{v} = \frac{D}{{S_{e} }}\vec{j}$ at the exit (either $\vec{v}_{*} = \frac{{S_{0} }}{{S_{e} }}\frac{D}{{D - D_{1} }}\vec{j}$ at the exit of the tank); $\vec{v} = \frac{{D_{1} }}{{S_{1} }}\vec{j}$ at the source of the pollutant–patient (either $\vec{v}_{*} = \frac{{S_{0} }}{{S_{1} }}\frac{{D_{1} }}{{D - D_{1} }}\vec{j}$ at the patient–the source of pollutant);

- for temperatures: $\theta = \theta_{0}$ at the inlet (i.e., $\theta_{*} = 0$ at the inlet in the tank); $\theta = \theta_{e}$ at the exit (i.e., $\theta_{*} = 1$ at the exit from the tank); $\theta = \theta_{0}$ to the patient–source of pollutant (either $\theta_{*} = \frac{{\theta_{1} - \theta_{0} }}{{\theta_{e} - \theta_{0} }}$ to patient–source of pollutant); $\theta = g_{1} \left( {x,y,z} \right)$ in the walls of the tank, where the coordinates *x, y, and z* describe the walls (let $\theta_{*} = g_{{1_{{}} }} \left( {x_{*} ,y_{*} ,z_{*} } \right)$ in the walls, where $g_{1*} \left( {x_{*} ,y_{*} ,z_{*} } \right)$ is a function that describes the walls);

- for concentrations: $c = c_{0}$ at the inlet (either $c_{*} = 0$ at the inlet in the tank); $c = c_{e}$ at the exit (either $c_{*} = \frac{{D_{1} }}{D}$ at the exit from the tank); $c = c_{1}$ to patient–source of pollutant (let $c_{*} = 1$ at the patient–source of pollutant);

- for densities: $\rho = \rho_{0}$ at the inlet (let $\rho_{*} = 1$ at the inlet in the tank); $\rho = \rho_{1}$ at the patient–source of pollutant (either $\rho_{*} = \frac{{\rho_{1} }}{{\rho_{0} }}$ at the source of pollutant). In the case of a stationary regime, the initial temporal conditions become null.

From the equations (5), (7), (10), and (15) and the boundary conditions, the following dimensionless groups are identified:

- $E = \frac{{v_{0}^{2} }}{{c_{p} \Delta \theta_{0} }}$ *Eckert* number (expresses the mass heat transfer);

- $$Re = \frac{{\rho v_{0} A}}{{\eta_{0} }} = \frac{{v_{0} A}}{\upsilon }$$ *Reynolds* number;

- $Pe = Re.Pr$ *Peclet* thermal number, with $Pr = \frac{{\eta_{0} c_{0} }}{\lambda }$ *Prandtl* number (expresses forced thermal convection);

- $Sc.Re$ *Peclet* mass number, with $Sc = \frac{\upsilon }{D}$ *Schmidt* number (expresses the diffusion);

- $Ar = \Delta \theta_{0} \beta \frac{gA}{{v_{0}^{2} }}$ *Archimedes* number; $Fr = \frac{{v_{0}^{2} }}{gA}$ *Froude* number;

$- \frac{{c_{1} }}{{\rho_{0} }}\left( {\frac{\partial \rho }{{\partial c}}} \right)$; $\frac{{S_{0} }}{{S_{1} }}\frac{{D_{1} }}{{D - D_{1} }}$; $\frac{{S_{0} }}{{S_{e} }}\frac{D}{{D - D_{1} }}$; $\frac{{\theta_{1} - \theta_{0} }}{{\theta_{e} - \theta_{0} }}$; $g_{1*}$; $\frac{{D_{1} }}{D}$; $\frac{{\rho_{1} }}{{\rho_{0} }}$ (expresses the geometric conditions).

Only those groups that respond to the way of formulating the problem are retained.

The indices* r* and *m* will be attached to the references to the real situation, respectively, model. The scale factor between the real case (the fluid in the real case is air) and the model is denoted by K and is given by the ratio $\frac{{A^{\left( r \right)} }}{{A^{\left( m \right)} }}$

#### The Case in which the Fluid is Oxygenated Air

In this case, the similarity conditions, expressed by Re *idem* and Ar *idem*, are the same. The similarity condition Ar *idem* is difficult to achieve from a practical point of view, which leads to the conclusion that the incompatibility will occur between the *Reynolds* condition and the *Archimedes* condition, which cannot be satisfied simultaneously. For this reason, basic modeling methods are used, which avoid the difficulties created by the practical incompatibility of similarity conditions (use of self-modeling domains, hydraulic, or geometric distortions). It has been shown that *Reynolds* number does not intervene in laws superior to a certain critical value (the field of self-modeling is used, which means certain conditions of production of the phenomenon in nature and on the model). The resemblance to *Reynolds* becomes

$Re> Re^{\left( m \right)}_{{{\mathrm{critic}}}}$ in the model tank, and $Re> Re^{\left( r \right)}_{{{\mathrm{critic}}}}$ in the real hyperbaric chamber

It is assumed that for such values of the number Re, the densities of the flow of matter and temperature are much higher than the densities of flow of matter and temperature determined by molecular diffusion. This hypothesis aims to remove the mass and thermal similarity of *Peclet*.

If the model works at a constant temperature (the *Archimedes* condition is removed), then the remaining dimensionless groups completely determine the development of the phenomenon:

16 $$\left( 1 \right)\,\frac{{c_{1} }}{{\rho_{0} }}\left( {\frac{\partial \rho }{{\partial c}}} \right)_{0} \,\left( 2 \right)\,Fr = \frac{{v_{0}^{2} }}{gA}\,\left( 3 \right)\,\frac{{S_{0} }}{{S_{1} }}\frac{{D_{1} }}{{D - D_{1} }}\,\left( 4 \right)\,\frac{{S_{0} }}{{S_{e} }}\frac{D}{{D - D_{1} }}\,\left( 5 \right)\,\frac{{D_{1} }}{D}\,\left( 6 \right)\,\frac{{\rho_{1} }}{{\rho_{0} }}$$

Ensuring kinematic similarity requires, in addition to the *Reynolds* conditions for the model and the real case, also the observance of the conditions of geometric similarity:

17 $$\left( {\frac{{S_{0} }}{{S_{e} }}} \right)^{\left( r \right)} = \left( {\frac{{S_{0} }}{{S_{e} }}} \right)^{\left( m \right)} ;\,\left( {\frac{{S_{0} }}{{S_{1} }}} \right)^{\left( r \right)} = \left( {\frac{{S_{0} }}{{S_{1} }}} \right)^{\left( m \right)}.$$

The similarities noted above with (3), (4), and (5) are equivalent, meaning that the observance of equality between the real case and the model reduces to the dimensionless groups:

18 $$(a)\,\frac{{c_{1} }}{{\rho_{0} }}\left( {\frac{\partial \rho }{{\partial c}}} \right)_{0} ;(b)\,\frac{{v_{0}^{2} }}{gA};\,(c)\,{\mathrm{Re}}> {\mathrm{Re}}_{critic} ;\,(d)\,\frac{{\rho_{1} }}{{\rho_{0} }};\,(e)\,\frac{{D - D_{1} }}{{D_{1} }}.$$

This last system, consisting of five dimensionless groups, completely determines the development of the oxygenation phenomenon, without the need for the whole set of equations and conditions written at the beginning of the paragraph.

### Model Validation

For implementing the model, we have to calculate the values of the variables mentioned in the previous section. To accurately replicate the physical processes occurring inside the hyperbaric chamber, especially in scenarios involving the release of harmful substances, certain boundary conditions must be considered. These include designated zones for fresh air supply (S_₀_), pollutant introduction (S_1_), and pollutant exhaust (S_e_), as well as the associated airflow dynamics: D, representing the mixture exhaust flow, and D₁, corresponding to the exhaled air emitted by the patient, which contains carbon dioxide (CO_2_) and other trace gases. Within the dimensionless formulation, D_₁_ is defined as a secondary airflow term that interacts with the clean oxygenated flow (D), reproducing the physical processes of pollutant dispersion and dilution inside the chamber. This formulation captures the contribution of exhaled air to the overall flow balance, while individual physiological variability, such as differences in age, metabolism, or respiratory rate, is not included in the present model. These conditions are essential for analyzing dispersion phenomena within the chamber and ensuring that the scaled model faithfully reflects real-world behavior.

Achieving complete similarity among Reynolds, Archimedes, and Froude numbers in a reduced-scale model is generally not feasible for low-speed, mixed-convection flows such as those encountered in hyperbaric chambers. This limitation is well recognized in hydraulic and ventilation modeling and is commonly addressed through self-modeling flow regimes, where the influence of certain dimensionless groups becomes negligible. In the present configuration, airflow velocities are sufficiently low and temperature differences are small enough that buoyancy effects contribute only weakly to the overall flow structure. Consequently, Reynolds similarity serves as the principal criterion governing dynamic similarity in the model. The Archimedes and Froude numbers, although theoretically relevant, exert minimal influence under nearly isothermal laboratory conditions. For this reason, the scaled model aims to reproduce the qualitative airflow patterns and pressure control dynamics, rather than to provide a fully dimensionally identical replica of a clinical hyperbaric chamber. This approach is consistent with established modeling practices for enclosed airflows and environmental control systems.

To implement the model, we must calculate the values of the variables defined in the previous section. Considering a real configuration of a hyperbaric chamber, we apply a scale factor of K = 10, resulting in the following model dimensions: A ^m^ = 12mm (height), A_o_^m^ = 220 mm (length), and A_1_^m^5 mm (width). In this context, the superscript “m” denotes parameters related to the model, while “r” refers to the real system.

From the similarity condition, (e) $\left( {\frac{{D - D_{1} }}{D}} \right)^{\left( m \right)} = \left( {\frac{{D - D_{1} }}{D}} \right)^{\left( r \right)}$ will result the flows of the pollutant - exhaled air ($D_{1}^{\left( m \right)} = 0,45 {\mathrm{l}}/{\mathrm{min}}$), and of the clean fluid-oxygenated air ($D^{\left( m \right)} = 8,5 {\mathrm{l}}/{\mathrm{min}}$).

The similarity condition (b) requires that the *Froude* number in the model be equal to the *Froude* number in the real case

19 $$\frac{{v_{0}^{\left( r \right)} }}{{gA^{\left( r \right)} }} = \frac{{v_{0}^{\left( m \right)} }}{{gA^{\left( m \right)} }}\, \Rightarrow \,v_{0}^{\left( r \right)} = \frac{{\left( {D - D_{1} } \right)^{\left( r \right)} }}{{S_{0}^{\left( r \right)} }} = 0,5 m/s,\,v_{0}^{\left( m \right)} = \frac{{v_{0}^{\left( r \right)} }}{K} = 0,1 m/s ,$$

where $\left( {D - D_{1} } \right)^{\left( m \right)} = 8,05 {\mathrm{l}}/{\mathrm{min}}$, as obtained from the similarity condition (b).

The similarity condition (c) must also be met in the real case and in the model. The *Reynolds* number in the real case and in the model is 96000 and 16100, respectively. Under similarity condition (a), the calculation of the ratio $\frac{\partial \rho }{{\partial c}}$arises when the pollutant is a gaseous mixture composed of a clean and a toxic fluid.

Let $V_{1}$denote the specific volume occupied by the toxic air; from the mass balance equation, we obtain

20 $$\rho \left( c \right) = \rho_{0} \left( {1 - V_{1} } \right) + \rho_{1} V_{1} , \, where:\;\rho_{1} = \frac{c}{{V_{1} }} \Rightarrow \rho \left( c \right) = \rho_{0} \left( {1 - \frac{c}{{\rho_{1} }}} \right) + c.$$

Differentiating with respect to the concentration *c*,we obtain the final relation:

$$\frac{\partial \rho }{{\partial c}} = 1 - \frac{{\rho_{0} }}{{\rho_{1} }},$$

which can be used when the patient removes a pollutant that is a mixture of gases.

## Structure and Function of the Pressure Regulation System in Hyperbaric Chambers

Hyperbaric chambers operate by increasing the atmospheric pressure around the patient, which enhances the amount of oxygen dissolved in the blood plasma according to Henry’s Law. This increased oxygen availability is essential for treating conditions such as carbon monoxide poisoning, chronic wounds, and decompression sickness. However, the benefits of HBOT can only be realized if the pressure within the chamber is meticulously controlled [43–45].

Modern hyperbaric chambers utilize advanced pressure regulation systems that include both manual and automated controls. These systems typically consist of high-quality pressure valves, sensors, and computerized control units that work together to maintain the desired pressure levels. The regulation process begins with the compression phase, where air or oxygen is introduced into the chamber, gradually increasing the pressure. This is followed by the treatment phase, where the pressure is held steady for a prescribed duration. Finally, the decompression phase carefully reduces the pressure back to normal atmospheric levels to prevent any adverse effects, such as decompression sickness.

The pressure regulation system must account for various factors, including the type of hyperbaric chamber (mono-place or multi-place), the treatment protocol, and individual patient requirements. In mono-place chambers, which are designed for single patients, the regulation process is relatively straightforward. However, in multi-place chambers, which can accommodate multiple patients simultaneously, the system must ensure uniform pressure distribution and account for the different oxygen needs of each patient.

By ensuring patient safety, optimizing therapeutic efficacy, maintaining operational efficiency, and providing adaptability and monitoring, these systems play a critical role in delivering effective hyperbaric treatments.

Hyperbaric oxygen therapy involves inhaling pure oxygen in a pressurized chamber or cabin. The effects of hyperbaric oxygen therapy are based on the laws of gas and the physiological and biochemical effects of hyperoxia. In processes where the parameter to be adjusted is the pressure of a gas, the compressibility of the gas must be considered if the pressure variations are greater than 5%.

According to Boyle’s law:

21 $$PV = {\mathrm{const}}$$

at a constant temperature, the pressure (*P*) and the volume of a gas (*V*) are inversely proportional. This is the basis of many effects of hyperbaric therapy, including a slight increase in room temperature during treatment, but also the phenomenon known as “squeezing.” It occurs when blockages in the Eustachian tube prevent the gas pressure from equalizing, causing painful compression of the gas in the middle ear.

According to Dalton’s law, in a mixture of gases (n representing the number of gases),

22 $$P = P_{1} + P_{2} + ... + P_{n}$$

each element exerts pressure proportional to the fraction of the total volume (partial pressure).

According to Henry’s law,

23 $$P = kC$$

at constant temperature, the amount of gas dissolved in a liquid or tissue, at saturation (*C*), varies in direct proportion to the partial pressure of the gas (*P*) which is in contact with the liquid or tissue. This law is the basis for increasing oxygen saturation in tissues in HBOT treatments.

The adiabatic flow of a perfect gas is given by the relation [46]:

24 $$Q = KSc\sqrt {2g\left( {P_{1} - P_{2} } \right)\gamma } { },$$

where *Q* is the gas flow, *K* is the flow coefficient, *S* is the strangulation section, *c* is the expansion coefficient, $\gamma$ is the specific mass of the gas, and $P_{1}$ and $P_{2}$ are the absolute pressures of the gas at the inlet and outlet, respectively, of the strangled conduct.

The flow resistance is given by the relation:

25 $$R = \frac{dP}{{dQ}}$$

and is determined from the curves established experimentally for each type of strangulation separately, curves that express the flow as a function of the pressure difference.

The gas capacity *C* of a tank/chamber is defined by the relation:

26 $$C = \frac{dM}{{dP}},$$

where *M* is the mass in the chamber and *P* is the pressure.

**Fig. 2:**
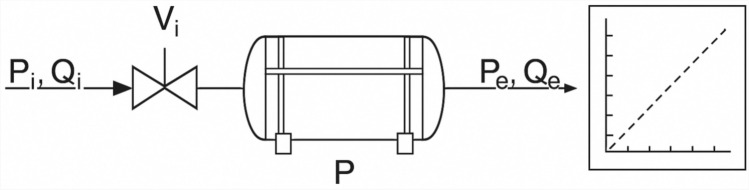
Explanatory schematic of the automatic gas pressure control system. The figure illustrates the controlled tank (hyperbaric chamber), inlet and outlet valves, and feedback loop maintaining the desired internal pressure. The model forms the basis for the subsequent analytical description of chamber pressurization dynamics

For practical cases and for processes taking place at usual pressures and temperatures, the capacitance C can be considered constant. The overall configuration and principal parameters of the modeled hyperbaric chamber are illustrated in Figure 2, which schematically represents the airflow inlet ($P_{i} ,Q_{i}$), outlet ($P_{e} ,Q_{e}$), and internal circulation paths used in the formulation of the governing equations. The operation of a process in which it is required to obtain a certain pressure in the tank is described by the following equation:

27 $$CdP = \left( {Q_{i} - Q_{e} } \right)dt,$$

where *P* is the gas pressure in the tank; *C* is the gas capacitance of the tank; $Q_{i}$ is the gas flow at the inlet; and $Q_{e}$ is the gas flow at the outlet.

In practice, the pressure drops are smaller than those that determine the supersonic flow, in which case the outlet flow $Q_{e}$ is a function of the pressure $P_{e}$ at the end of the outlet conduct:

28 $$Q_{e} = f_{e} \left( {P,P_{e} } \right).$$

Usually, the normal operation of such a process takes place around values $P = P_{0}$ of the pressure in the vessel and $P_{e} = P_{e0}$ of the outlet pressure. If the small variations of the parameters around the normal operating point are noted in lower case,

29 $$P = P_{0} + p,\;Q_{i} = Q_{i0} + q_{i} ,\;Q_{e} = Q_{e0} + q_{e} ,\;P = P_{e0} + p_{e}.$$

Keeping in mind that $Q_{e0} = Q_{i0}$:

30 $$C\frac{dp}{{dt}} = q_{i} - q_{e}.$$

From the relation (28), we can deduce

31 $$q_{e} = \left( {\frac{{\partial f_{e} }}{\partial P}} \right)_{0} p + \left( {\frac{{\partial f_{e} }}{{\partial P_{e} }}} \right)_{0} p_{e}.$$

**Fig. 3:**
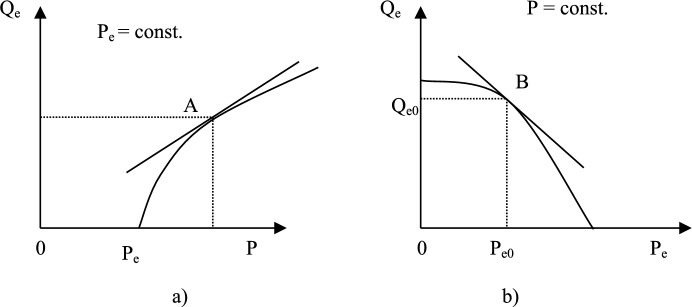
Variation of the outlet airflow $Q_{e}$ as a function of internal tank pressure $P$ and outlet pressure $P_{e}$. The curve illustrates how back pressure and outlet conductance affect gas discharge during hyperbaric operation

The curves that provide $Q_{e}$ as a function of $P$, for constant $P_{e}$ (Figure 3a), and $Q_{e}$ as a function of $P_{e}$, for constant $P$ (Figure 3b), were obtained experimentally for the specific case considered. The index “0” in expression (16) refers to the normal operating point, denoted by points A and B (Figure 3a,b). The following notations are introduced:

32 $$\left( {\frac{{\partial f_{e} }}{\partial P}} \right)_{0} = \frac{1}{R\beta };\left( {\frac{{\partial f_{e} }}{{P_{e} }}} \right)_{0} = - \frac{1}{{R_{e} }}.$$

The resistance $R_{e} \left[ \frac{1}{ms} \right]$ was introduced at the outlet flow, and the relation (30) becomes with these notations:

33 $$q_{e} = \frac{1}{R\beta }p - \frac{1}{{R_{e} }}p_{e}$$

The resulting variation of outlet flow with pressure is shown in Figure 3. A similar relationship can be defined for the inlet valve, where

34 $$Q_{i} = f_{i} \left( {M_{v} ,P_{i} ,P} \right).$$

For small variations around the normal point $P = P_{0}$ of operating, noting, just like in the previous case, $M_{v} = M_{v0} + m$ ; $P_{i} = P_{i0} + p_{i}$ ; $P = P_{0} + p$, it results:

For small variations around the normal operating point $P = P_{0}$, and using the same notation as in the previous case, namely $M_{v} = M_{v0} + m$, $P_{i} = P_{i0} + p_{i}$, and $P = P_{0} + p$, the following relation is obtained:

35 $$q_{i} = \left( {\frac{{\partial f_{i} }}{{\partial M_{v} }}} \right)_{0} m + \left( {\frac{{\partial f_{i} }}{{\partial P_{0} }}} \right) + \left( {\frac{{\partial f_{i} }}{\partial P}} \right)p.$$

By introducing the coefficient $C_{v} \left[ {\frac{kg}{{ms}}} \right]$, characteristic of the inlet valve, as well as the flow resistances along the inlet conduit, the relation becomes

36 $$q_{i} = C_{v} m + \frac{1}{{R_{i} }}p_{i} - \frac{1}{{R_{\alpha } }}p,$$

where $R_{\alpha } \left[ \frac{1}{ms} \right]$ is the absolute flow resistance on the inlet pipe corresponding to $P = P_{0}$ . By introducing the values of $q_{e}$ and $q_{i}$ from the relations (32) and (33) in the relation (29), we obtain

37 $$C\frac{dp}{{dt}} = C_{v} m + \frac{1}{{R_{i} }}p_{i} - \frac{1}{{R_{\alpha } }}p + \frac{1}{{R_{e} }}p_{e} - \frac{1}{{R_{\beta } }}p,$$

or

38 $$T\frac{dp}{{dt}} + p = K_{m} m + K_{e} p_{e} + K_{i} p_{i},$$

where the time constant of the process is $T = \frac{{R_{\alpha } R_{\beta } C}}{{R_{\alpha } + R_{\beta } }}$, and $K_{m} = \frac{{R_{\alpha } R_{\beta } C_{v} }}{{R_{\alpha } + R_{\beta } }}$, $K_{e} = \frac{{R_{\alpha } R_{\beta } }}{{R_{e} \left( {R_{\alpha } + R_{\beta } } \right)}}$,$K_{i} = \frac{{R_{\alpha } R_{\beta } }}{{R_{i} \left( {R_{\alpha } + R_{\beta } } \right)}}$

This process is described by a first-order differential equation. The time constant *T* of the process depends, in addition to the capacity *C* of the tank, also on the resistances $R_{\alpha }$ and $R_{\beta }$ which have different values ​​according to the point $P_{0}$ in which the operation of the process takes place. In equation (37), the coefficients *T*, $K_{m}$, $K_{e}$, and $K_{i}$ can only be considered for small variations around the normal operating values. If the pressure adjustment in the tank is done by acting on the position of the stem of a valve $V_{e}$ located downstream and assuming the flow in the supersonic valve, meaning the ratio $\frac{{P_{e} }}{{P_{0} }} < 0,53$ (for air), and therefore, the flow $Q_{e}$ does not depend on the $P_{e}$, then the operation of the process can be described by the relationships:

39 $$C\frac{dp}{{dt}} = q_{1} - q_{e} ,\,\;q_{i} = \frac{1}{{R_{i} }}p_{i} - \frac{1}{{R_{\alpha } }}p,\;q_{e} = C_{v} m + \frac{1}{{R_{\beta } }}p$$

from which the equation is deduced

40 $$T\frac{dp}{{dt}} + p = - K_{m} m + K_{i} p_{i},$$

where *T* is the time constant of the process having the same expression as before.

The transfer function of the process, considering as input quantity the variation of the flow produced by the opening of the valve $V_{e}$, is therefore of the form $\frac{K}{Ts + 1}$ . Since the relationships must be linear, in order to obtain the transfer functions of the process, the problems of pressure regulation must have some similarities with those of level regulation.

## Laboratory Implementation and Testing of the Pressure Regulation System

The experiments conducted within the scope of hyperbaric oxygen therapy (HBOT) are designed to achieve multiple objectives, each aimed at enhancing the understanding and efficacy of this medical treatment. The primary goals of these experiments include validating the mathematical models of oxygen and pressure dynamics, optimizing treatment protocols, and ensuring patient safety and comfort. By validating mathematical models, optimizing treatment protocols, and focusing on patient safety and comfort, these experiments contribute to the continual improvement and wider acceptance of HBOT as a critical therapeutic modality.

### Experimental Setup

When air is inhaled at normal values of pressure (1 atmosphere), the blood pressure is about 100 mmHg, and the oxygen pressure in the tissues is about 55 mmHg. A saturation of 100% with oxygen, at 3 atmospheres, can increase blood pressure to 2000 mmHg and tissue oxygen tension to about 500 mmHg, allowing the release of 60 ml of oxygen per liter of blood (compared to 3 ml/l at normal atmospheric pressure), enough to support the function of poorly oxygenated tissues without a contribution of oxygen from hemoglobin.

**Fig. 4:**
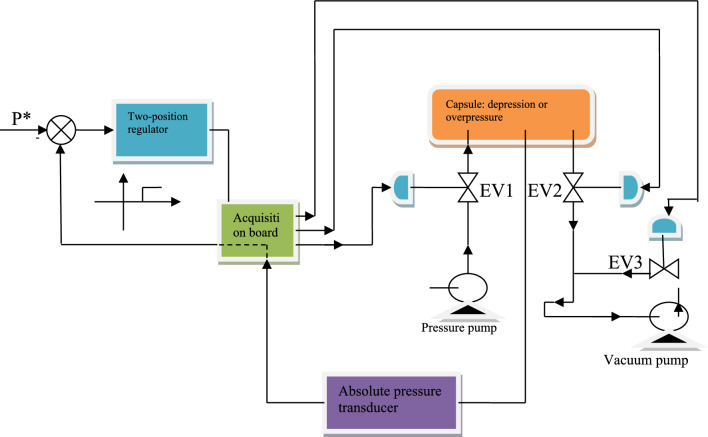
Functional diagram of the automatic pressure control system implemented in the experimental hyperbaric chamber. The system includes an absolute pressure transducer, three solenoid valves (EV1–EV3), a pressure pump, and a vacuum pump operating in a closed-loop configuration to maintain the desired chamber pressure

The proposed system (Figure 4) was developed to regulate the absolute pressure within a hermetically sealed hyperbaric chamber. It integrates sensors, actuators, and control elements that operate in a feedback loop to maintain stable chamber pressure during both pressurization and decompression phases.

The EV1 solenoid valve, when in the deactivated position, blocks the passage of gaseous fluid into the enclosure. It is controlled to increase the absolute pressure within the enclosure. Simultaneously, the EV2 solenoid valve is closed to prevent the removal of gaseous fluid from the enclosure by the vacuum pump. This prevents overloading of the drive motor and reduces wear on the vacuum pump.

The vacuum pump has triple the flow compared to the pressure pump, allowing for rapid achievement of vacuum in the range of 0 to 1.5 atmospheres, approximately half the time needed for achieving overpressure in the same range.

Control of the three solenoid valves, as well as starting and controlling the electric drive motor of the two pumps, is managed by the computer through an acquisition board. This board includes an analog input channel for reading signals from the MPX 5100 AP absolute pressure transducer, along with four digital output channels.

The system operates as follows: a reference value for absolute pressure is set as a reference from the graphical interface.

If the pressure in the chamber falls below the reference value, the command is issued to start the two pumps and control solenoid valves EV1 and EV3 to increase the chamber pressure. Conversely, if the pressure in the enclosure exceeds the reference value, the command is issued to control the EV2 solenoid valve to reduce the pressure. When the pressure in the enclosure equals the reference value, all pumps and solenoid valves are stopped.

In a hyperbaric oxygen therapy chamber, the air pressure is increased to three times the normal air pressure. Under these conditions, a significantly higher concentration of pure oxygen is absorbed into the lungs compared to normal air pressure. This increased oxygen concentration is then transported through the bloodstream to all parts of the body. The elevated systemic oxygen levels aid in combating bacteria and stimulate the synthesis and release of growth factors and stem cells, promoting tissue regeneration and healing.

In animal experiments, a mouse is positioned within a hermetically sealed chamber where pure oxygen is supplied at a pressure 1.5 times higher than atmospheric pressure. This process enhances the body’s healing and recovery capabilities by elevating oxygen levels in the bloodstream, resulting in hyperoxygenation of the body.

### Implementation of the pressure regulation system on a laboratory model

**Fig. 5:**
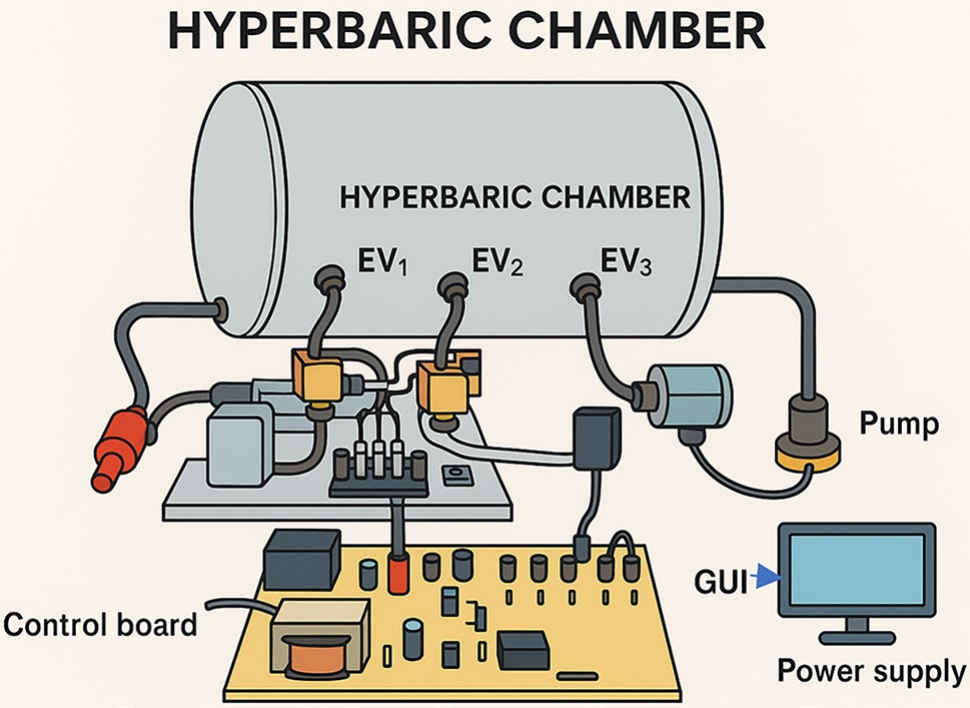
General view of the laboratory-scale hyperbaric chamber model used for experimental studies. The setup reproduces the geometric proportions and functional configuration of a clinical hyperbaric chamber, allowing controlled investigation of pressure regulation and airflow behavior under laboratory conditions

**Fig. 6:**
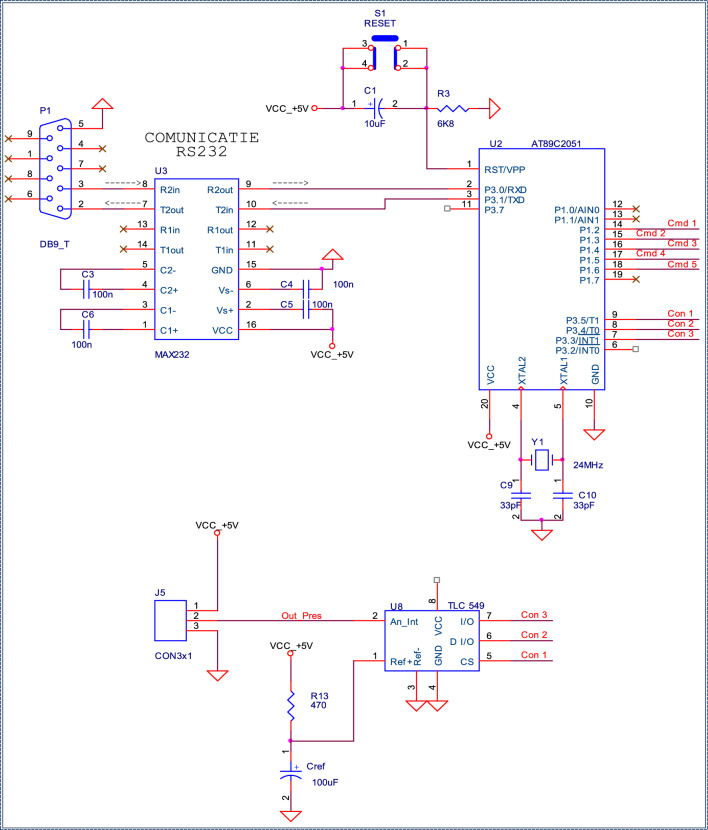
Representation of the acquisition and control board based on the ATMEL AT89C52 microcontroller. The board incorporates a quartz oscillator, reset, and serial communication modules, providing real-time control and data acquisition for the hyperbaric pressure regulation system

To validate the automatic pressure-adjustment functionality of the experimental system, a laboratory-scale model was designed and implemented (Figure 5). The setup includes two volumetric pumps, one for pressurization and one for vacuum, driven by a common electric motor. The control and data-acquisition unit, presented in figure 6, is centered on an ATMEL AT89C52 microcontroller.

Although the prototype employs an AT89C52 microcontroller and legacy acquisition hardware, this choice does not limit the validity or applicability of the proposed control architecture. The regulation algorithm, valve actuation logic, and sensor feedback scheme are fully platform-independent and may be implemented without modification on modern embedded platforms such as STM32 microcontrollers, Arduino/ARM-based development boards, FPGA systems, or NI industrial controllers. The hardware used in the present study was selected for laboratory feasibility and component availability; however, the underlying principles, dynamic response characteristics, and system topology remain scalable and compatible with current standards in biomedical engineering instrumentation. This ensures that the technological relevance of the work extends beyond the specific hardware configuration employed in the prototype.

Figure 6 presents the acquisition and control board developed around the ATMEL AT89C52 microcontroller. The board integrates the key peripheral components required for data collection and process control, including an 11.0592 MHz quartz oscillator, a reset circuit, and a serial communication interface that connects the hardware to the host computer.

The microcontroller programming process follows standard sequential steps: selecting the target memory address via the address lines, entering the corresponding data, activating the control-signal combination, applying a high state to $\overline{EA} /V_{pp}$ for programming, and generating an $ALE/\overline{PROG}$ pulse to write the bit. Each write cycle is internally timed and completes within approximately 1.5 ms.

The microcontroller communicates with the computer, respectively, with the processor according to a protocol presented in Table 1.

**Table 1:** Transmission protocols defining the bidirectional communication between the microcontroller and the host processor, including data request and command frames

Transmission		
No. byte	Value	Significance
01	T	T
02	C	C
03	Value	Order issued at the port
04	32	CRC
01	T	T

Reception		
No. byte	Value	Significance
01	T	T
02	D	Data demand
03	D	D
04	32	CRC

Transmission		
No. byte	Value	Significance
01	T	T
02	D	D
03	Value	Data from the convertor
04	32	CRC

Reception		
No. Byte	Value	Significance
01	T	T
02	D	C
03	Value	Order issued at the port
04	32	Crc

This communication protocol operates through two distinct four-byte frames exchanged bidirectionally between the host computer and the microcontroller (AT89C2051). Each frame follows the generic format [T, X, Y, CRC], where

- T represents the frame type identifier (transmission header),
- X defines the instruction type (D—data request or C—command),
- Y contains either the analog measurement or the digital command value, and
- CRC is the cyclic redundancy check byte ensuring transmission integrity.

When the microcontroller receives a frame of the form [T, D, D, CRC], the second byte (D) is interpreted as a request for process data. In response, the microcontroller returns a frame [T, D, ADC_value, CRC], where the third byte contains the digital value produced by its analog-to-digital converter (ADC). Thus, whenever byte 2 = D, the system performs an analog measurement (e.g., pressure sensor reading) and transmits the corresponding digital value to the computer.

Conversely, when the frame [T, C, Command, CRC] is received, the second byte (C) instructs the microcontroller to update its digital outputs. The third byte (Command) specifies the bit pattern to be written to Port P0, which drives the system’s actuators—three solenoid valves (EV1–EV3) and the start/stop module of the motor powering the pumps.

The output port of the computer is connected to the control inputs of the three solenoid valves and to the start/stop circuit of the electric motor driving the two pumps. The control logic follows a standard digital pattern, where a logic “1” disables the valve coil and a logic “0” activates it.

After executing the command, the microcontroller returns a confirmation frame of identical structure, [T, C, Command, CRC], acknowledging successful transmission and output update. In this way, every instruction sent by the computer is both executed and verified through feedback communication.

The logic of the output control is as follows:

- When a bit of Port P0 is set to logic “1,” the corresponding transistor Q conducts, pulling the gate of the IRF540 MOSFET to ground. This switches the MOSFET off and de-energizes the associated solenoid coil.
- When the output bit is logic “0,” transistor Q is cut off, the MOSFET gate receives its bias voltage, and the solenoid coil becomes energized, opening the valve.

This bidirectional protocol ensures synchronized control between the host computer and the hyperbaric system’s actuators while maintaining communication reliability through CRC verification.

**Fig. 7:**
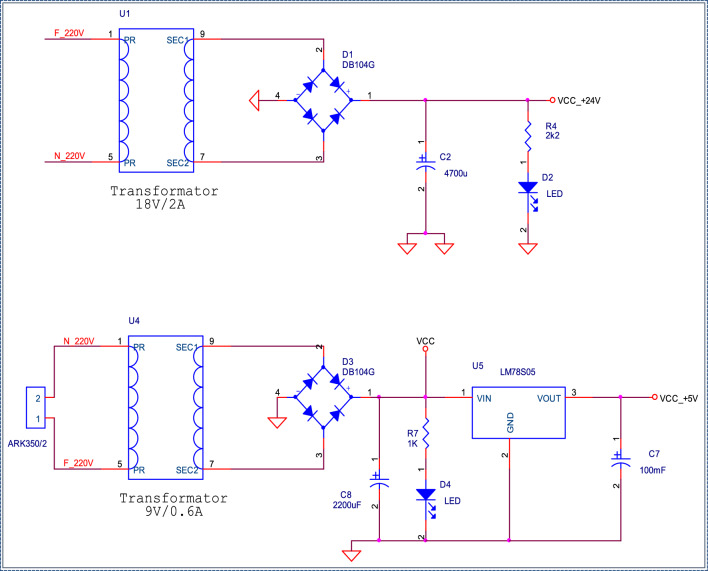
Wiring diagram of the power supply for the acquisition and control board. The circuit provides regulated voltages for the microcontroller and solenoid valve drivers

Figure 7 illustrates the electrical circuit supplying power to the acquisition and control board, as well as the source driving the solenoid valves. The system follows a conventional design, incorporating a 220/5 V or 220/24 V transformer, bridge rectifiers, filter capacitors, and voltage-stabilization modules to ensure reliable operation of the control electronics and actuators.

When a mouse is introduced into the laboratory model, such as a pressurized hyperbaric chamber, and inhales oxygen at a concentration of 97%, with a flow rate of approximately 8 liters per minute, it stimulates the body’s natural healing mechanisms. Pressurization typically takes place over a period of 10 to 30 minutes, followed by depressurization lasting between 4 and 10 minutes. The pressurization process can be reversed at any point. Generally, a complete treatment regimen involves a minimum of 10 sessions, each lasting between 60 and 90 minutes.

The high-concentration oxygen inhaled during pressurization, consisting of molecules smaller than hemoglobin molecules, dissolves into all body fluids (including lymphatic, cerebrospinal, and plasma), easily penetrating capillaries and areas with poor vascularization, no longer relying solely on hemoglobin for transport.

### Limitations of the experimental validation

The validation performed in this study focuses primarily on assessing pressure stabilization, control reproducibility, and airflow behavior within a scaled hyperbaric environment. These tests confirm the operational feasibility of the proposed pressure regulation system but do not encompass full characterization of the oxygen distribution or internal flow fields. Oxygen concentration mapping, flow visualization, and computational fluid dynamics (CFD) comparisons were beyond the scope of the current work and remain planned for future development.

Our objective was not to reproduce full patient physiology or clinical oxygen transport, but rather to evaluate the chamber’s mechanical and control characteristics under defined laboratory conditions. The scaled model is designed to support incremental validation steps, where additional sensors, high-resolution flow measurement techniques, and CFD-based analyses can be integrated in subsequent research phases.

Furthermore, the present experiments were conducted under isothermal and simplified boundary conditions to ensure stability and repeatability. Although these conditions facilitate the evaluation of control behavior, they do not fully capture the thermodynamic complexity of operational HBOT chambers. Future work will extend the validation framework to include transient thermal effects, multi-point oxygen sampling, and detailed comparison with numerical simulations.

Another limitation of the current model is that it does not explicitly account for the composition or variability of exhaled gases, particularly carbon dioxide (CO_2_) and humidity, which can influence the internal air mixture within clinical hyperbaric chambers. These parameters differ depending on patient age, metabolic activity, and respiratory patterns. Future work will aim to integrate these physiological factors through extended gas–flow coupling and dynamic diffusion models to improve the realism of the simulations.

## Conclusions

The study presented in this paper has addressed critical aspects of hyperbaric oxygen therapy (HBOT), emphasizing the importance of mathematical modeling, pressure regulation, and experimental validation in enhancing the efficacy and safety of this therapeutic approach.

First, we highlighted the significance of cutting-edge technologies and advanced modeling techniques in optimizing the hyperbaric environment. Mathematical models provide invaluable insights into the oxygen dynamics and pressure variations within hyperbaric chambers. These models not only facilitate the design and implementation of efficient HBOT protocols but also contribute to the development of safer and more comfortable treatment environments for patients.

Second, the research underscored the pivotal role of precise pressure regulation in hyperbaric chambers. Proper pressure management is essential for achieving the therapeutic goals of HBOT while minimizing the risks of adverse effects such as barotrauma and decompression sickness. The integration of sophisticated control systems and real-time monitoring ensures that the pressure levels within the chamber remain within safe and effective limits.

Third, the experiments conducted as part of this study have validated theoretical models and optimized treatment protocols. By comparing experimental data with model predictions, we have been able to refine our understanding of the physiological responses to HBOT. These findings support the development of evidence-based treatment guidelines that can be tailored to individual patient needs, thereby enhancing the overall efficacy of HBOT.

Furthermore, the study explored the therapeutic benefits of HBOT across a range of medical conditions. Our findings confirm that HBOT significantly improves tissue oxygenation, promotes angiogenesis, accelerates wound healing, and enhances immune response. The ability of HBOT to mobilize bone marrow stem cells and generate new neural cells underscores its potential in treating neurological conditions and promoting overall health.

Additionally, the study examined the implications of HBOT on cancer treatment, revealing that intermittent exposure to hyperbaric oxygen does not stimulate tumor growth. This finding is crucial for the safe integration of HBOT into multimodal cancer therapies, including radiation and chemotherapy.

Lastly, the practical implementation of the pressure regulation system on a laboratory model demonstrated the feasibility and reliability of the proposed system. This step is vital for translating theoretical models into real-world applications, ensuring that HBOT remains a viable and effective therapeutic option.

The pressure control behavior of the proposed laboratory model demonstrates stable regulation and repeatability, comparable in principle to that of commercial HBOT chambers, though achieved on a reduced scale. Commercial systems typically employ industrial-grade proportional valves, redundant sensors, and certified safety modules to ensure fine-grained pressure ramping and automatic emergency shutdown. In contrast, the present prototype uses solenoid-based actuation and a single-sensor feedback loop, suitable for research and educational purposes rather than direct clinical use. Nevertheless, the implemented control algorithm accurately reproduces the pressurization and decompression cycles of clinical systems, validating the theoretical model and enabling safe experimentation under controlled conditions. The methodology developed here can therefore inform future upscaling of HBOT designs by integrating redundant sensors, improved materials, and certified safety interlocks.

The mathematical simulations revealed that moderate changes in pressurization rate and chamber pressure strongly influence oxygen diffusion efficiency. For instance, a 10% increase in chamber pressure was associated with an approximate 8–9% improvement in modeled oxygen partial pressure (pO₂) within the chamber environment, consistent with reported physiological responses in tissue oxygenation. Such relationships demonstrate that accurate control of pressure dynamics can optimize therapeutic oxygen delivery, thereby supporting the biomedical relevance of the proposed model.

In conclusion, this research advances our understanding of hyperbaric oxygen therapy by integrating mathematical modeling, experimental validation, and practical implementation. The insights gained from this study contribute to the optimization of HBOT protocols, ultimately improving patient outcomes and expanding the therapeutic potential of this modality. Future research should continue to explore the nuances of oxygen dynamics, refine pressure regulation techniques, and validate findings in clinical settings to further enhance the benefits of HBOT.
